# Efficacy of Ultrasound-Guided Injections of Type I Collagen-Based Medical Device for Greater Trochanteric Pain Syndrome: A Pilot Study

**DOI:** 10.3390/life15030366

**Published:** 2025-02-26

**Authors:** Filippo Randelli, Alberto Fioruzzi, Manuel Giovanni Mazzoleni, Alessandra Radaelli, Leila Rahali, Lucia Verga, Alessandra Menon

**Affiliations:** 1 Hip Department–CAD, ASST Gaetano Pini–CTO, Piazza Cardinale Andrea Ferrari 1, 20122 Milano, Italy; filippo.randelli@asst-pini-cto.it (F.R.); manuelmazzoleni4@gmail.com (M.G.M.); 2 Scuola di Specializzazione in Ortopedia e Traumatologia, Università degli Studi di Milano, Via Festa del Perdono 7, 20122 Milano, Italy; alessandra.radaelli8@gmail.com; 3 UOC Radiodiagnostica, ASST Gaetano Pini–CTO, Piazza Cardinale Andrea Ferrari 1, 20122 Milano, Italy; leila.rahali@asst-pini-cto.it (L.R.); lucia.verga@asst-pini-cto.it (L.V.); 4 ASST Gaetano Pini–CTO, Piazza Cardinale Andrea Ferrari 1, 20122 Milano, Italy; ale.menon@me.com

**Keywords:** hip pain, collagen injections, tendinopathy, ultrasonography, interventional, regenerative medicine

## Abstract

**Background:** Greater Trochanteric Pain Syndrome (GTPS) is a frequent clinical condition characterized by acute or chronic pain in the lateral region of the hip. This condition is primarily due to gluteus minimus and medius tendinopathy. Swine-derived type I collagen has shown a positive effect on tenocytes through in vitro studies and on tendinopathies in clinical studies. This pilot study aims to evaluate the clinical effects of swine-derived type I collagen injections on pain, hip function, and strength in GTPS patients. **Methods:** The study group was treated with three ultrasound-guided swine-derived type I collagen injections once a week for three consecutive weeks. The primary endpoint was pain reduction of at least 3 points on the Numeric Rating Scale (NRS) at ten weeks. Secondary endpoints were NRS average reduction at rest and palpation, modified Harris Hip Score (mHHS), abductor strength, and magnetic resonance imaging (MRI) improvement at six months. **Results:** 52 patients were screened, 47 enrolled, and 43 completed the study. The primary endpoint was reached by 60.5% of the patients. All secondary endpoints were also reached with statistical significance. Neither early nor late adverse effects were found. **Conclusions:** In this pilot study, ultrasound-guided peritrochanteric swine-derived type I collagen injections are safe and effective for most patients with GTPS included in the study. Further and more extensive confirmatory investigation studies with a longer follow-up are needed to confirm this pilot study’s results and the clinical benefit’s persistence.

## 1. Introduction

Greater Trochanteric Pain Syndrome (GTPS) is a frequent and complex clinical condition characterized by acute or chronic and more or less relapsing pain in the lateral region of the hip around the greater trochanter [1,2].

In the past, lateral hip pain was attributed exclusively to inflammation of the peritrochanteric bursa, which was described as “trochanteric bursitis” [3,4,5,6].

GTPS, instead, can result from different mechanical and inflammatory pathologies involving the peritrochanteric structures, which are formally divided into three primary entities: external snapping hip syndrome, trochanteric bursitis, and, more frequently, degenerative gluteus minimus and medius muscle tendinopathies [3,4,5,6].

The treatment of GTPS is initially conservative and includes rest, ice, non-steroidal anti-inflammatory drugs (NSAIDs), and physical therapy focusing on postural exercises and iliotibial band stretching [7,8,9]. The second line of treatment is constituted by infiltrative therapy, classically through the use of steroids but presently more driven towards the use of orthobiologics, primarily platelet-rich plasma (PRP). The use of steroids, unfortunately, does not appear to be effective in the medium–long term [7,10]. Few studies have been conducted with PRP in recent years without definitive evidence of its efficacy and safety [11,12,13,14,15]. Most studies either fail to demonstrate consistent benefits or are impaired by methodological issues such as small sample sizes, short follow-up periods, or biases [16,17]. Furthermore, PRP injections are expensive.

Swine-derived type I collagen is a new entry in the panorama of orthobiologics but has demonstrated a good scientific basis. In two in vitro studies [11,12], the effects of swine-derived type I collagen on a culture of human tenocytes were described, focusing on collagen turnover pathways to understand how this could improve tendon biology. Porcine collagen is similar to human collagen and highly compatible; it presents very low risks of inducing adverse effects, so it has been used in different clinical settings [11,12]. Researchers used swine-derived type I collagen as a coating for cell cultures of human tenocytes harvested from a gluteus minimus tendon sample collected during hip surgeries [11,12]. The results suggested that swine-derived type I collagen can promote synthesis, maturation, and secretion of human type I collagen (COL-I), thus positively regulating tendon homeostasis. Furthermore, swine-derived type I collagen-induced an anabolic phenotype in tenocytes by stimulating their proliferation and migration [11,12].

Few pilot and observational clinical studies on using swine-derived type I collagen in tendinopathies, such as supraspinatus tendinopathy and lateral epicondylitis, reported significant pain reduction and functional recovery with a strong safety profile [13,15,18,19,20].

Can swine-derived type I collagen be beneficial in treating greater trochanteric pain syndrome (GTPS)? The primary objective of the present pilot study is to answer this fundamental question.

The primary endpoint of this study was to evaluate pain reduction, at least 3 points on the NRS scale at T10w/FU in patients affected by GTPS treated with injectable swine-derived type I collagen medical device.

Secondary endpoints were NRS of pain, pre- and post-procedural, at rest at 6 and 24 weeks; mHHS, pre- and post-procedural at 6, 10, and 24 weeks; abduction strength, pre- and post-procedural at 6, 10, and 24 weeks; painkiller consumption during the time study; and evaluation of phlogistic and degenerative signs through MRI images at 24 weeks. Furthermore, dropouts and adverse events were recorded.

## 2. Materials and Methods

The study is a single-center pilot clinical investigation with a one-sample design. It was approved by the Institutional Review Board (protocol code OSMAMI-14/05/2021-0021380-U, dated 14 May 2021). The National Clinical Trial Number (NCT) is 05486078.

The subjects were selected among patients who met the inclusion criteria, did not meet the exclusion criteria, and were willing to sign the informed consent.

Male and female subjects aged between 18 and 70 who met the following criteria were considered eligible: palpatory lateral pain lasting at least one month; NRS pain level ≥ 5 in the trochanteric area; capability to collaborate, understand, and sign in the informed consent.

The exclusion criteria were the following: concomitant intra-articular hip pain (with positive flexion–adduction–internal rotation test); ESHS (External Snapping Hip Syndrome); total hip replacement in the affected hip; radiological and clinical evidence of gluteus minimus and/or medius tendons tears with an indication for surgical repair; evidence of tendon calcifications documented radiographically; hip osteoarthritis (Tönnis classification > 1); fluoroquinolones treatment within 30 days before enrollment; hip injection with hyaluronic acids or steroids within four weeks before the enrollment; local or systemic infection; chronic treatment with steroids or immunosuppressants; drug and/or alcohol addiction, psychiatric disorders or clinical conditions that may compromise the correct interpretation of Patient-Reported Outcome Measures (PROMs) or follow-up; coagulopathies, platelet aggregation disorders, or treatment with oral anticoagulants or antiplatelets that cannot be suspended during the study period; pregnancy and breastfeeding; allergy to porcine collagen.

Pre-procedural (T0) diagnostic exams included a Pelvic X-ray and Dunn 45° axial views of the hip, as well as a high-field MRI 1.5 T with different sequences: coronal T1 and STIR (FAT suppression), axial T1, and STIR.

### 2.1. Medical Device (Swine-Derived Type I Collagen)

This study used an injectable medical device based on swine-derived type I collagen extracted from the dermis, GUNA MD-Tissue (GUNA S.p.a. Milan, Italy). The product, already marketed under the form of 2 mL/vials, contains 100 µg of collagen per vial together with ^1^ Magnesium gluconate, ^2^ Ascorbic acid, ^2^ Pyridoxine hydrochloride, ^2^ Riboflavin, ^2^ Thiamine hydrochloride, and as auxiliary substances, NaCl and Water WFI. The auxiliary substances were supplied by ^1^ Merck Life Science S.r.l. Milan, Italy and ^2^ A.C.E.F. S.p.a. Fiorenzuola d’Arda (PC), Italy.

### 2.2. Technique

The all-study group was treated with three ultrasound-guided injections of 2 mL of GUNA MD-Tissue—one injection every seven days for three consecutive times.

The sono-anatomy of the greater trochanteric region is crucial for accurate ultrasound-guided procedures in Greater Trochanteric Pain Syndrome (GTPS). The trochanteric bursa appears as a thin hypoechoic band between the tensor fascia lata (TFL) and the gluteus medius tendon at the lateral facet of the greater trochanter, with bursitis presenting as a fluid-filled, thickened structure. The gluteus medius tendon, triangular in shape, inserts onto the lateral aspect of the greater trochanter. In contrast, the gluteus minimus tendon attaches to the anterior facet as a flat, hyperechoic structure. The iliotibial band (ITB) runs superficially over the greater trochanter, and the bone cortex should appear as a continuous hyperechoic line, with any irregularities suggesting chronic tendinopathy. Dynamic ultrasound assessment, such as passive hip movements and compression maneuvers, helps differentiate bursal effusion from intra-tendinous edema [21].

The injections were guided by an ultrasound device (MyLab™ XPRO 80, Esaote S.p.a., Genova, Italy). The procedures were performed with the patient in a lateral decubitus. After preparing a sterile field, a linear (15–18 MegaHertz) probe was placed longitudinally along the gluteus tendons, precisely at the most painful trigger point and where the most significant area of microstructural inhomogeneity was visible. At that point, a 20–22 Gauge spinal needle was inserted under constant ultrasound guidance [22]. MD-Tissue was injected into the trochanteric bursa and at the level of the tendons, particularly at the most degenerated insertional areas.

### 2.3. Study Visits Timeline

The subjects were evaluated at six different times: at baseline, before the first treatment (time T0), after one week (T1w), after two weeks (T2w), after six weeks (T6w/FU), after ten weeks (T10w/FU), and after 24 weeks (T24w/FU), when two highly qualified musculoskeletal radiologists performed a post-procedural Pelvic MRI for each patient’s further radiological evaluation.

Pain assessment was conducted through NRS at rest and during the more specific clinical tests for GTPS. Patients were asked to rate the pain level at rest on a scale of 0 to 10, with 0 indicating no pain and 10 representing the worst imaginable pain. Additionally, pain was assessed during palpation over the greater trochanter with the patient in the lateral decubitus. This procedure is the standard clinical practice for diagnosing and monitoring GTPS. Both types of pain assessments—at rest and during palpation—were performed before treatment and at each follow-up visit.

mHHS was obtained before treatment and then at each follow-up visit.

Hip abductor strength was assessed with the subject supine using a dynamometer (Kern HCB 20K10, Kern & Sohn GmbH, Ebingen, Germany) with a maximum weighing capacity of 20 kg and a reproducibility of 0.01 kg, with a 30 s interval between measurements to avoid muscle fatigue and ensure consistency, according to the technique described by Thorborg [23]. The mean obtained by three repeated measurements was used for statistical analysis.

Two expert radiologists evaluated pre-procedural and post-procedural MRI images, focusing on perilesional edema in axial and coronal STIR-weighted images.

### 2.4. Statistical Analysis

Given the one-sample study design, it is assumed that the proportion of successes (patients experiencing a reduction of at least 3 points on the NRS scale) without treatment cannot surpass 25% (H0). Conversely, a success rate of at least 50% (HA) is expected in treated patient subjects.

With these premises, a one-tailed exact binomial test applied to a sample of 49 subjects reaches a power of 95.7% in discriminating the difference equal to 25% between the proportion predicted by HA and that predicted by H0 at a significance level equal to 0.025.

Possible dropout subjects cannot determine sample size inflation, as each subject of this type will automatically be considered a failure.

All the variables were subjected to the appropriate descriptive analysis after validation. According to distribution, continuous variables were presented as mean and standard deviation or median and range. Categorical variables were expressed in terms of case numbers or frequencies. Statistical analyses were conducted with Stata/SE 17.0 (StataCorp LLC, College Station, TX, USA).

The primary endpoint was assessed with a one-tailed exact binomial test. It was evaluated at T10w/FU, and the NRS score at T10w/FU was compared to T0. Reducing the NRS scale by 3 points in at least 50% of the treated subjects was considered clinically significant.

The within-subject repeated measures ANOVA or Friedman test was used to evaluate the NRS score, mHHS score, and abductor strength at T6w/FU and T24w/FU compared to time T0.

The Student’s *t*-test for paired data or Wilcoxon test was used to assess the evidence of resolution or decrease in inflammatory signs in the peri-trochanteric region of the hip affected according to MRI images at T24w/FU compared to T0.

The repeated measures ANOVA or Friedman test was used to evaluate painkiller consumption based on the clinical diary at T0, T1w, T2w, T6w/FU, T10w/FU, and T24w/FU.

The absolute and relative frequency and 95% confidence interval of the fraction of subjects who abandoned the study concerning Adverse Events were also reported.

## 3. Results

**Figure 1:**
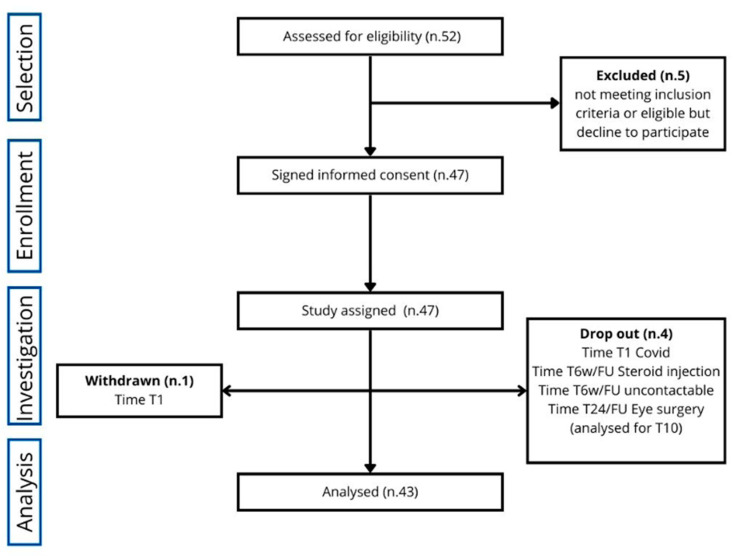
CONSORT study flowchart.

Of the initially eligible 52 subjects, 47 were enrolled in the study for 13 months, from October 2021 to November 2022. Of these 47 patients, one withdrew at time T1w, and four dropped out (one at T1w, two at T6w/FU, and one at T24w/FU), as illustrated in Figure 1. Therefore, the subjects who completed the study were 42, even though the subjects analyzed for the primary endpoint were 43. The study population consisted of individuals with a mean age of 53.72 years (SD ± 10.3 years), reflecting a middle-aged demographic with moderate variability in age. The sample predominantly comprised females, who accounted for 89% of the participants. This gender distribution highlights a significant female predominance; these characteristics suggest that the study population represents a targeted group. No adverse effects were recorded during the study period.

### 3.1. Primary Endpoint—Pain Reduction at Rest of at Least 3 Points at 10 Weeks

**Figure 2:**
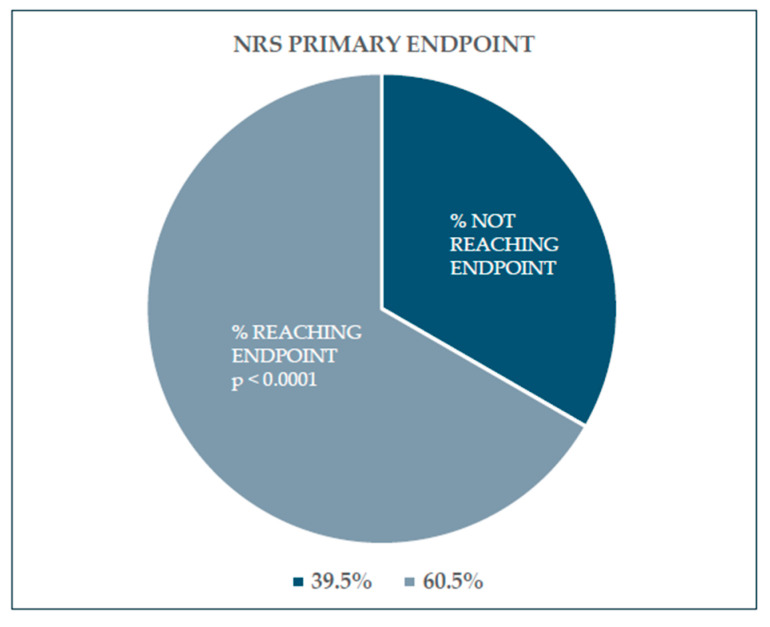
The study’s primary endpoint. At the end of the survey, 60.5% of the enrolled subjects reached the primary endpoint (*p* < 0.0001), and 39.5% did not reach the primary endpoint despite having reduced pain.

At T10w/FU, 26 patients, representing 60.5% of the participants, experienced a significant reduction in pain—specifically, a decrease of more than 3 points on the NRS. These patients met the criteria for the study's primary endpoint. Conversely, 17 patients (39.5%), despite having had a reduction in pain, did not achieve the primary endpoint (Figure 2). In conclusion, the study’s primary endpoint has to be considered complete.

### 3.2. Secondary Endpoints

#### 3.2.1. NRS of Pain at Rest, at Baseline, 6 Weeks, 10 Weeks, and 24 Weeks

**Figure 3:**
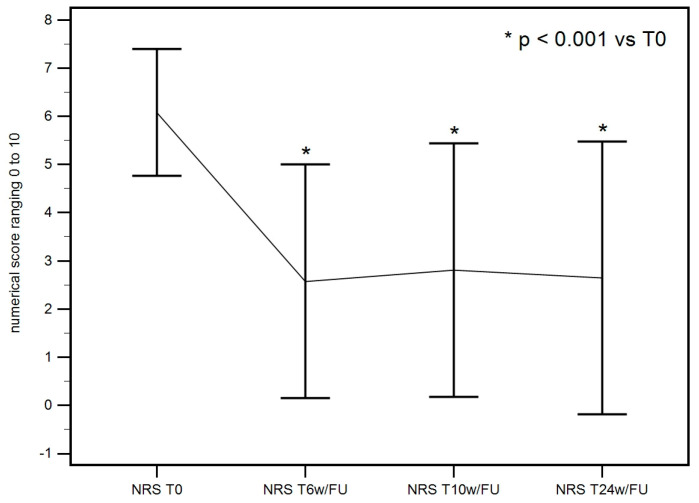
NRS of pain at rest at baseline, 6 weeks, 10 weeks, and 24 weeks descriptive plot. A significant pain reduction was achieved at T6w/FU and maintained at T10w/FU and T24w/FU.

A statistically significant improvement in pain at rest was reached at 6 weeks and maintained till 24 weeks. The F-test for within-subject variation was significant (*p* < 0.001) under any assumption regarding sphericity. Pairwise tests corrected with the Bonferroni method show that the values at T0 significantly differed from those at T6w/FU, T10w/FU, and T24w/FU (*p* < 0.001 in all cases), as seen in Table 1 (Figure 3).

#### 3.2.2. NRS of Pain on Palpation at Baseline, 6 Weeks, 10 Weeks, and 24 Weeks

**Figure 4:**
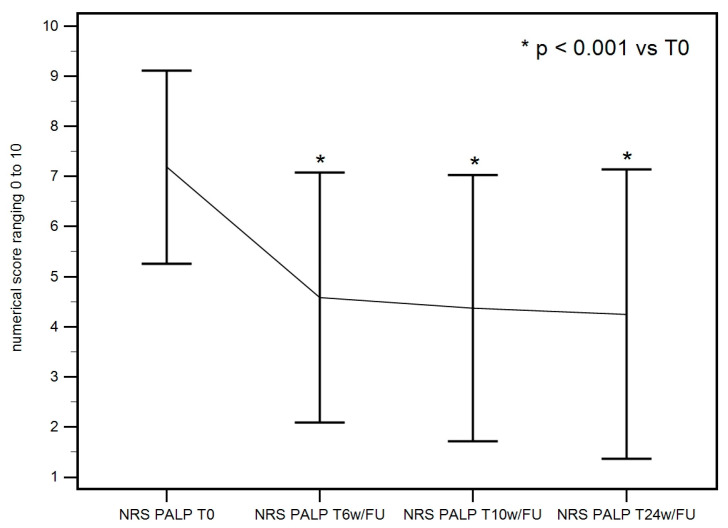
NRS of pain on palpation on palpation at baseline, 6 weeks, 10 weeks, and 24 weeks descriptive plot. A significant pain reduction on palpation was achieved at T6w/FU and maintained at T10w/FU and T24w/FU.

Collected data for pain on palpation showed a statistically significant improvement at 6 weeks, which was maintained at 10 and 24 weeks. The F-test for within-subject variation was significant (*p* < 0.001) for any sphericity assumption. The Pairwise tests corrected with the Bonferroni method show that the values at T0 significantly differ from those at T6w/FU, T10w/FU, and T24w/FU (*p* < 0.001 in all cases), as seen in Table 2 (Figure 4).

#### 3.2.3. mHHS at Baseline, 6 Weeks, 10 Weeks, and 24 Weeks

**Figure 5:**
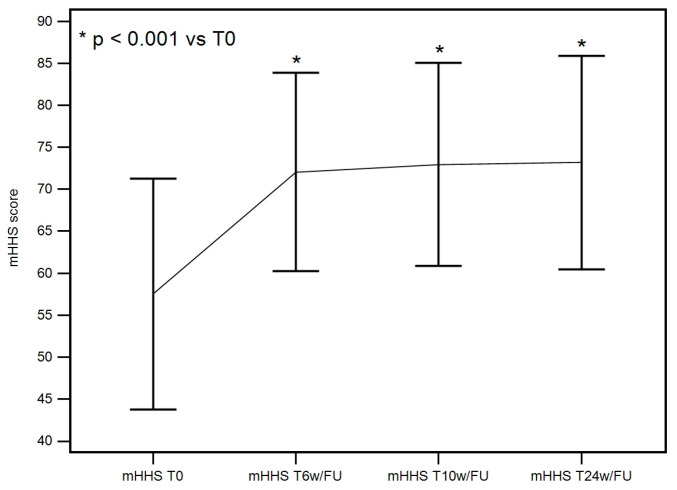
mHHS at baseline, 6 weeks, 10 weeks, and 24 weeks descriptive plot. A significant improvement of mHHS was achieved at T6w/FU and maintained at T10w/FU and T24w/FU.

A statistically significant improvement in mHHS was achieved at 6 weeks and maintained at 10 and 24 weeks. The F-test for within-subject variation was significant (*p* < 0.001) under any assumption regarding sphericity. Pairwise tests corrected with the Bonferroni method show that the values at T0 significantly differed from those at T6w/FU, T10w/FU, and T24w/FU (*p* < 0.001 in all cases), as seen in Table 3 (Figure 5).

#### 3.2.4. Abduction Strength at Baseline, 6 Weeks, 10 Weeks, and 24 Weeks

**Figure 6:**
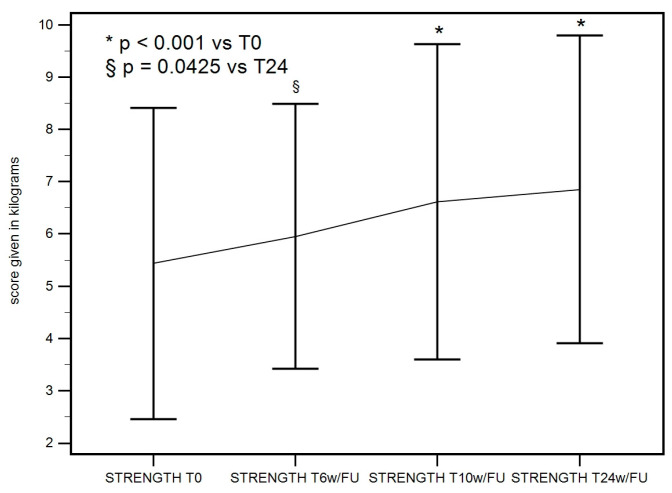
Abduction strength at baseline, 6 weeks, 10 weeks, and 24 weeks descriptive plot. A significant improvement in abduction strength was recorded at T10w/FU and maintained till T24w/FU.

A statistically significant improvement in strength in hip abduction was recorded at 10 weeks and maintained till 24 weeks. The F-test for within-subject variation was significant (*p* < 0.001) for any sphericity assumption. Pairwise tests corrected with the Bonferroni method show that the values at T0 were significantly different compared to those at T10w/FU (*p* < 0.001) and T24w/FU (*p* < 0.001), as seen in Table 4 (Figure 6). A slightly significant difference (*p* = 0.0425) was observed between T6w/FU and T24w/FU.

#### 3.2.5. Painkiller Consumption During the Study Time

Painkiller consumption did not significantly increase during the study (Friedman test: *p* = 0.992). However, in two patients, between T10w/FU and T24w/FU, an abnormal increase in painkiller consumption was observed, going from 1 to 12 and from 1 to 13, respectively.

#### 3.2.6. MRI Evaluation at 24 Weeks Compared to Baseline

**Figure 7:**
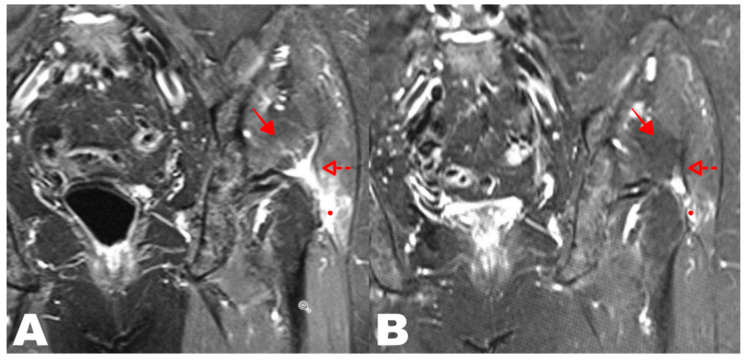
(**A**) Pre-treatment MRI coronal STIR sequence of a patient’s left hip affected by GTPS with the presence of a fluid signal in the insertional area of the gluteus medius tendon; (**B**) MRI coronal STIR sequence of the same hip at T24s/FU with an apparent reduction of fluid. The full point indicates the bursal effusion; intra-tendinous edema is indicated by the dotted arrow, and muscle edema is indicated by the full arrow.

After six months, comparable MRI images of the peritrochanteric area were available for 40 out of 43 subjects analyzed. In 28 patients (70%), a reduction in peri-tendinous edema was visible by MRI at T24w/FU compared to T0 (Figure 7), while in 12 patients (30%), no changes were found (see MRI data table). The McNemar exact test was statistically significant, as seen in Table 5 (*p* < 0.0001).

## 4. Discussion

This study suggests that ultrasound-guided swine-derived type I collagen injections are a safe and effective alternative for Greater Trochanteric Pain Syndrome (GTPS), offering longer-lasting pain relief and functional improvement compared to corticosteroids. MRI-based tendon changes indicate potential regenerative effects, positioning collagen as a viable orthobiologic option. Given its safety and accessibility, collagen therapy could enhance GTPS management, though more extensive randomized trials are needed to confirm its long-term benefits.

Lateral peritrochanteric pain, defined as Greater Trochanteric Pain Syndrome (GTPS), according to Karpinski (1985) [24], is mainly due to a minimus and medius glutei tendinopathy and represents one of the most frequent causes of pain in the hip area. Various treatments have been proposed, but no one has shown a clear superiority. The orthopedic community has recently moved from classic treatments (e.g., physiotherapy, focal shock wave therapy, and steroid injections) to a new approach focused on restoring biological homeostasis. Ultrasound-guided injections of platelet-rich plasma (PRP), bone marrow aspirate [25], autologous tenocytes [26], and percutaneous fenestration procedures [16] are examples of this trend.

A 2021 systematic review [27] of randomized controlled trials, which analyzed 1034 patients, demonstrated that PRP and focal shock waves significantly improve pain reduction. Unfortunately, the authors also showed that neither of the two therapies maintained these clinical improvements.

A 2023 Bayesian analysis [28] of 596 GTPS patients treated with ultrasound-guided infiltrations of either PRP or corticosteroids and focal shock waves showed that PRP was the most efficacious pain reduction agent, followed by shock waves. The study demonstrated that both therapies were safe and effective, but neither the abduction strength nor the MRI imaging were analyzed.

In a single-blinded, double-arm, randomized controlled trial, Heaver C. et al. [29] compared the results of focal shock wave therapy with corticosteroids ultrasound-guided infiltrations. The primary endpoint was pain reduction. At one year follow-up, the group treated with focal shock waves showed a higher statistically significant improvement than those treated with corticosteroids. This study also demonstrated an improvement in the Trendelenburg sign. The improvement was maintained over time in the group treated with focal shock waves but not in the group treated with corticosteroid injections.

Focused extracorporeal shockwave therapy (fESWT) is widely used for Greater Trochanteric Pain Syndrome (GTPS) due to its ability to promote tendon healing and pain relief through various biological mechanisms [30,31]. It stimulates tenocyte proliferation, collagen synthesis, and extracellular matrix remodeling, enhancing tendon regeneration. fESWT also promotes neovascularization, improving oxygen and nutrient supply while reducing inflammation by modulating cytokine activity. Additionally, it decreases pain sensitivity by downregulating substances P and CGRP, breaks down calcific deposits, and activates mesenchymal stem cells, fostering tissue repair [32]. These effects make fESWT a promising, non-invasive alternative for GTPS management. However, some observed benefits from collagen injections may stem from the mechanical stimulation of the tendon rather than collagen’s biochemical properties, similar to ultrasound-guided fenestration. Further controlled studies are needed to distinguish collagen’s specific role from mechanical effects.

Over the last decade, promising evidence has arisen from studies on extracellular matrix (ECM) scaffolds [17] in functional tissue engineering. Swine-derived type I collagen has an excellent scientific basis in in vitro studies [11,12] and some evidence of its effectiveness in clinical practice for musculoskeletal disorders [13,14,15,33]. It has been used for treating epicondylitis [17], plantar fasciitis [34], knee osteoarthritis [35], supraspinatus tendinopathy [13], and myofascial pain [36].

The main aim of the present study was to evaluate the effectiveness of ultrasound-guided peritrochanteric injections of swine-derived type I collagen (MD-Tissue, GUNA S.p.a. Milan, Italy) for GTPS.

The study evaluated pain reduction at rest through the NRS scale at 10 weeks compared to baseline in 43 subjects affected by GTPS (primary endpoint).

Some secondary endpoints were evaluated: NRS of pain at rest at 6 weeks and 24 weeks and NRS of pain on palpation, mHHS, and abduction strength at baseline at 6 weeks, 10 weeks, and 24 weeks.

All of the abovementioned secondary endpoints achieved statistical significance.

Besides the first battery of secondary endpoints, painkiller consumption during time study and MRI evaluation were investigated.

The increase in painkiller use during follow-up could be due to individual variability in treatment response, as some patients may have an initial local transient inflammatory response to the injection. Additionally, GTPS symptoms can fluctuate, with periods of improvement followed by flare-ups, potentially prompting higher analgesic use. Increased physical activity after initial pain reduction may have also led to temporary exacerbations, while psychological factors, such as patient expectations, could have influenced pain perception and medication reliance. Furthermore, tendon healing is gradual, and some patients may still experience discomfort as the tissue adapts. Lastly, comorbidities or other musculoskeletal conditions, such as osteoarthritis or lumbar spine issues, could have contributed to pain, making it necessary for some individuals to increase painkiller consumption despite undergoing treatment.

Among the secondary endpoints, two need to be highlighted.

The first was hip strength in abduction, which slowly and progressively increased up to 24 weeks after treatment. However, it is impossible to know whether this trend was due to pain reduction or, as supposed, a real biological tendon improvement. The myotendinous junction and tendon fibers take time to regain physiological functionality, mimicking the same trend we could observe.

MRI evaluation at 24 weeks compared to baseline revealed that 70% of patients exhibited decreased peri-tendinous fluid accumulation. Notably, the fluid signal on MRI was primarily associated with intra-tendinous edema rather than bursal effusion, as the imaging analysis focused on changes within the gluteus minimus and medius tendons. This distinction is essential, as intra-tendinous edema suggests ongoing tendon pathology and biological response to treatment, whereas bursal effusion is typically linked to inflammatory processes. The reduced intra-tendinous edema observed in most cases may indicate a positive tissue remodeling effect following swine-derived type I collagen injections. However, it is worth noting that imaging improvements did not always correlate with clinical pain reduction, underscoring the complexity of GTPS pathophysiology and the multifactorial nature of pain perception. Future studies incorporating quantitative MRI analysis could further elucidate the relationship between structural changes and clinical outcomes.

The lack of correlation between peritendinous edema on MRI and pain in chronic tendinopathy is well documented, as the primary pain generators are neo-vessels and neo-nerves infiltrating degenerated tendon tissue. This has been extensively studied in other tendons, such as the common extensor tendon in lateral elbow tendinopathy, using high-resolution ultrasound [37]. Given this evidence, a sonographic follow-up rather than an MRI could be considered in future studies to assess tendon microvasculature changes before and after collagen injections. Future research should prioritize high-resolution ultrasound assessments to evaluate the microvascular and neural alterations in chronic tendinopathy, particularly after collagen injections. Given that neo-vessels and neo-nerves are key contributors to tendinopathy-related pain, ultrasound techniques such as power Doppler and superb microvascular imaging (SMI) could offer greater sensitivity in detecting pathological vascularization compared to MRI. This would allow a more functional and real-time analysis of tendon changes, improving our understanding of treatment effects on pain modulation and regeneration. Implementing longitudinal sonographic follow-ups could provide new insights into the healing process, enabling better patient selection and individualized treatment protocols for GTPS.

No other study has previously evaluated the effect of an injectable collagen-based medical device administered with ultrasound-guided injections as a therapy for GTPS. This study focused on quantitatively assessing the abductor strength and qualitatively investigating it through MRI.

The cohort’s mean age, 53.4 years, is a strength of this study. In fact, the higher prevalence is between 40 and 60 years of age [37].

This study has several major and minor limitations that should be acknowledged in line with its nature as a pilot study. Significant limitations include the absence of a control group, limiting the ability to compare collagen injections with other treatments such as corticosteroids or platelet-rich plasma (PRP). Additionally, the small sample size and single-center design reduce the generalizability of the findings. The relatively short follow-up period (24 weeks) prevents conclusions about the long-term durability of treatment effects. Minor limitations include potential selection bias, as the study population consisted primarily of middle-aged females, which may not fully represent all GTPS patients.

Furthermore, MRI findings did not always correlate with clinical improvements, raising the need for more precise imaging biomarkers to assess tendon healing. Future studies should focus on randomized controlled trials (RCTs) with larger sample sizes and multicenter participation to validate these findings. Investigating long-term outcomes beyond 24 weeks will help determine the persistence of pain relief and functional recovery. Additionally, further research should explore collagen injections’ biomechanical and histological effects on tendon regeneration. Comparative studies evaluating collagen injections against corticosteroids, PRP, or other orthobiologic treatments could provide insights into the most effective and cost-efficient approach. Lastly, developing standardized ultrasound protocols for optimal injection techniques and treatment monitoring will improve reproducibility and clinical adoption.

Another potential limitation of this study is that at least part of the observed therapeutic effect may be attributed to the mechanical stimulation of the tendon tissue rather than solely to the biological action of the swine-derived type I collagen. The ultrasound-guided injection technique, which involved delivering collagen into the bursa and directly into the tendon, could have had a mechanical effect on the degenerated tendon fibers. This is comparable to ultrasound-guided fenestration (dry needling), a widely recognized technique for treating chronic tendinopathy, where repeated needle penetrations stimulate the tendon’s healing response by inducing localized bleeding, fibroblast activation, and collagen synthesis. The flow of the injected mixture within the tendon may have further contributed to this mechanical effect, triggering a cascade of biological responses, including tenocyte activation and neoangiogenesis, independent of the collagen’s biochemical properties. While this does not diminish the potential benefit of collagen injections, it raises the need for future controlled studies comparing collagen injections with fenestration alone to distinguish the specific contribution of collagen from the mechanical effects of the injection technique itself.

Despite the discussed limitations, this pilot study, thanks to a rigorous and careful methodology, has shown that swine-derived type I collagen can represent a possible tool to treat GTPS without adverse effects and more than satisfactory preliminary results.

## 5. Conclusions

This pilot study suggests that ultrasound-guided swine-derived type I collagen injections are a safe and effective treatment for Greater Trochanteric Pain Syndrome (GTPS), significantly reducing pain and improving hip function and strength with no adverse effects. Pain reduction was achieved in 60.5% of patients at 10 weeks and sustained at 24 weeks, while MRI findings indicated structural tendon benefits in 70% of cases. Compared to corticosteroids and PRP, collagen injections appear to offer long-term advantages. However, the absence of a control group and limited follow-up warrant further large-scale trials to confirm the efficacy and long-term benefits.

## Figures and Tables

**Table 1:** NRS descriptive statistics.

NRS SUMMARY STATISTICS TABLE		
Evaluation Times	Mean	SD
NRS T0	6.154	1.3480
NRS T6w/FU	2.711	2.4250
NRS T10w/FU	2.860	2.6239
NRS T24w/FU	2.643	2.8334

**Table 2:** NRS of pain on palpation descriptive statistics.

NRS PALP SUMMARY STATISTICS TABLE		
Evaluation Times	Mean	SD
NRS PALP T0	7.277	1.8624
NRS PALP T6w/FU	4.633	2.4895
NRS PALP T10w/FU	4.337	2.6384
NRS PALP T24w/FU	4.250	2.8929

**Table 3:** mHHS descriptive statistics.

mHHS SUMMARY STATISTICS TABLE		
Evaluation Times	Mean	SD
mHHS T0	57.085	13.780
mHHS T6w/FU	71.222	11.9162
mHHS T10w/FU	72.535	12.2637
mHHS T24w/FU	73.190	12.7418

**Table 4:** Abduction strength descriptive statistics.

ABDUCTION STRENGHT SUMMARY STATISTICS TABLE		
Evaluation Times	Mean	SD
STRENGHT T0	5.538	2.8413
STRENGHT T6w/FU	6.045	2.4797
STRENGHT T10w/FU	6.676	3.0115
STRENGHT T24w/FU	6.851	2.9481

**Table 5:** MRI McNemar test.

MRI DATA TABLE			
40 Subjects Evaluated	Improved	Unchanged	*p* Value
MRI T24w/FU vs. T0	28 subjects (70%)	12 subjects (30%)	*p* < 0.0001

## Data Availability

All the data are available upon reasonable request. The corresponding author will share the article’s data upon reasonable request.

## Abbreviations

GTPS	Greater Trochanteric Pain Syndrome
NRS	Numeric Rating Scale
mHHS	Modified Harris Hip Score
MRI	Magnetic Resonance Imaging
PRP	Platelet-Rich Plasma
NSAIDs	Non-Steroidal Anti-Inflammatory Drugs
ECM	Extracellular Matrix
RCT	Randomized Controlled Trial
FU	Follow-Up
T0, T1w, T2w, T6w, T10w, T24w	Time points (Baseline, 1 week, 2 weeks, 6 weeks, 10 weeks, 24 weeks)
COL-I	Type I Collagen
ESHS	External Snapping Hip Syndrome
GCP	Good Clinical Practices
STIR	Short Tau Inversion Recovery (MRI Sequence)
T1	T1-Weighted MRI Imaging
Tönnis Classification	System for grading hip osteoarthritis
MD	Tissue—Swine-derived type I collagen medical device (GUNA S.p.A.)
IRB	Institutional Review Board
OSMAMI	Milano Area 2 Ethics Committee Code
NCT	National Clinical Trial Number
